# Proton Pump Inhibitor Therapy Does Not Affect Prognosis of Cirrhosis Patients With Acute Decompensation and Acute-on-Chronic Liver Failure: A Single-Center Prospective Study

**DOI:** 10.3389/fmed.2021.763370

**Published:** 2021-11-10

**Authors:** Shanshan Sun, Wenyi Ye, Ruihong Zhao, Jianhua Hu, Xuan Zhang, Meifang Yang, Hong Zhao, Jifang Sheng

**Affiliations:** ^1^State Key Laboratory for Diagnosis and Treatment of Infectious Diseases, National Clinical Research Center for Infectious Diseases, Collaborative Innovation Center for Diagnosis and Treatment of Infectious Diseases, The First Affiliated Hospital, Zhejiang University School of Medicine, Hangzhou, China; ^2^Department of Traditional Chinese Internal Medicine, The First Affiliated Hospital of Zhejiang Chinese Medical University, Hangzhou, China

**Keywords:** proton pump inhibitor, acute decompensated cirrhosis, acute-on-chronic liver failure, prognosis, complications

## Abstract

**Background:** The aim of this study was to investigate the impact of proton pump inhibitor (PPI) therapy on complications and prognosis in cirrhosis patients with and without acute-on-chronic liver failure (ACLF).

**Materials and Methods:** Cirrhosis patients with acute decompensation (AD) (*n* = 489) admitted in our center were enrolled in this prospective observational cohort study. According to treatment received, patients were identified as users or nonusers of PPI. Clinical and laboratory data, complications during hospitalization, and overall survival were recorded in all the patients.

**Results:** Of the 489 patients, 299 (61.1%) patients received PPI therapy. The logistic regression analysis showed that age, albumin, history of previous hepatic encephalopathy (HE), and the chronic liver failure-sequential organ failure assessment (CLIF-SOFA) score were independent risk factors for HE in patients with decompensated cirrhosis [odds ratio (OR) = 1.07, 95% CI: 1.03–1.12, *p* = 0.001; OR = 1.13, 95% CI: 1.04–1.24, *p* = 0.006; OR = 242.52, 95% CI: 40.17–1464.11, *p* < 0.001; and OR = 2.89, 95% CI: 2.11–3.96, *p* < 0.001, respectively]. Previous severe liver injury and previous bacterial infections were independent risk factors for spontaneous bacterial peritonitis (SBP) in patients with decompensated cirrhosis (OR = 3.43, 95% CI: 1.16–10.17, *p* = 0.026 and OR = 6.47, 95% CI: 2.29–18.29, *p* < 0.001, respectively). The multivariate Cox proportional hazards regression model showed that the type and dose of the PPI used were not related to 28-day and 90-day mortality in cirrhosis patients with AD or ACLF.

**Conclusion:** PPI use does not appear to increase mortality or the risk of HE and SBP in the hospitalized cirrhosis patients with and without ACLF.

## Introduction

Proton pump inhibitors (PPIs) are commonly used in cirrhosis patients to treat gastrointestinal disorders, especially gastrointestinal bleeding (1–3). Gastrointestinal hemorrhage may be due to bleeding varices or ulcers or portal hypertensive gastropathy and is one of the most serious complications seen in acute-on-chronic liver failure (ACLF) (4–6). While PPI is useful in these cases, the drug may alter the composition of the gut microbiota and increase the risk of spontaneous bacterial peritonitis (SBP), hepatic encephalopathy (HE), *Clostridium difficile* infection, and mortality (7–14). Although some recent studies have indicated that PPI therapy does not increase the risk of SBP or mortality in cirrhosis, these studies did not include patients with ACLF (15–18). The aim of this study was to assess whether PPI therapy increases the incidence of SBP and HE and decreases survival in cirrhosis patients with and without ACLF.

## Materials and Methods

### Patients

Patients diagnosed as cirrhosis with acute decompensation (AD) and hospitalized in the Department of Infectious Diseases at the First Affiliated Hospital, School of Medicine, Zhejiang University, Zhejiang, China, between February 2014 and March 2015 were eligible for inclusion in this study. Patients were excluded if they: (1) were <18 years old; (2) were pregnant; (3) had evidence of infection with human immunodeficiency virus, Epstein-Barr virus, or human cytomegalovirus; (4) had aplastic anemia, myelodysplastic syndrome, thrombocytopenia, hemophilia, or disseminated intravascular coagulation; (5) were currently using immunosuppressant medications or adrenocortical hormones; (6) had hepatocellular carcinoma or other malignant tumors; (7) had chronic renal disease or other serious comorbidity; or (8) had history of liver transplantation (19, 20). The diagnosis of liver cirrhosis was based on clinical evidence, endoscopic or radiologic examination, and liver biopsy (6, 21). Cirrhosis patients were diagnosed with AD if they had more than one of the following major complications: ascites, encephalopathy, gastrointestinal hemorrhage, severe liver injury, and infection (20). Severe liver injury was diagnosed if alanine aminotransferase (ALT) was ≥ five times the normal upper limit or more than twice the baseline value along with elevation of serum bilirubin to ≥ 85 μmol/l and international normalized ratio (INR) to ≥ 1.5 at any time during the preceding 1 month (21). The chronic liver failure-sequential organ failure assessment (CLIF-SOFA) score was used to diagnose organ failure (6). The diagnosis of ACLF was based on the criteria proposed by the Chinese Group on the Study of Severe Hepatitis B (COSSH) (22). The diagnosis of gastrointestinal bleeding and bacterial infections was made as previously described (1, 23, 24). PPI treatment was generally used in patients with gastrointestinal bleeding and gastric ulcer, but was extended in patients receiving endoscopic variceal ligation and those manifesting gastrointestinal disturbances such as hiccups, epigastric pain, nausea, or vomiting. For this study, “PPI use” was defined as intravenous administration of any PPI for at least 6 days with daily dose higher than that recommended by the WHO (13). Patients receiving lower doses were considered to be nonusers. PPI doses are classified according to the cumulative defined daily dose (cDDD) (25).

Informed consent for participation in this study was obtained from each participant or his or her legal surrogate. This study was approved by the Ethics Committee of the First Affiliated Hospital, School of Medicine, Zhejiang University, Zhejiang, China and all the procedures were in accordance with the latest version of the Declaration of Helsinki.

### Data Collection

Demographic data, clinical history, reason for admission, physical examination findings, laboratory results, cirrhosis complications, events of organ failure, and prognosis were recorded. Potential precipitating factors for SBP and HE were noted. Survival at 28 days and 90 days following enrollment was recorded. Survival information was obtained by telephonic interview.

### Statistical Analysis

The Statistical Package for the Social Sciences (SPSS) version 26.0 (IBM Corporation, Armonk, New York, USA) was used for the data analysis. Categorical variables were summarized as numbers and percentages and compared by using the chi-squared test. Continuous variables were summarized as means ± SDs or medians (and interquartile ranges) and compared by using the Student's *t*-test or the Mann–Whitney *U* test. Variables found to be associated with SBP and HE on the univariate analysis (*p* < 0.10) were entered into the multivariate logistic regression analysis to identify the independent risk factors for SBP or HE. Baseline characteristics were compared between patients receiving and not receiving PPI therapy before and after propensity score matching (PSM). We performed PSM to adjust differences in baseline characteristics of decompensated cirrhosis and ACLF including gender, age, leukocyte count, platelet count, hemoglobin, serum bilirubin, albumin, aspartate aminotransferase (AST), ALT, INR, creatinine, serum sodium, the CLIF-SOFA score, and the model for end-stage liver disease (MELD) score by matching non-PPI users with comparable patients with PPI users in a ratio of 1:1 with a caliper of 0.1 of the SD. A standardized difference < 0.1 indicated a good balance between PPI and non-PPI groups. The multivariate Cox proportional hazards regression model was used to analyze the risk factors for 28-day and 90-day mortality. For the multivariate analysis, the probability for stepwise entry was at *p* = 0.05 and for removal at *p* = 0.10; only variables with *p* < 0.05 were retained in the final model. Statistical significance was at *p* ≤ 0.05.

## Results

### Basic Characteristics of PPI Users and Non-users

**Figure 1:**
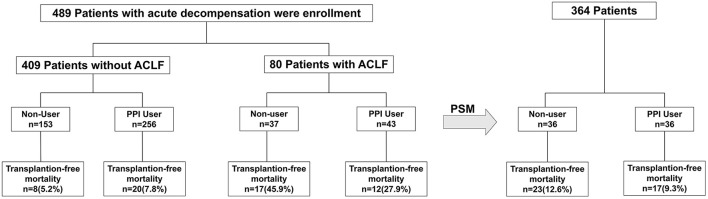
Flowchart showing patient selection for the study. A total of 489 acute decompensated patients with liver cirrhosis were selected and then propensity score matched with 1:1. PPI, proton pump inhibitor; ACLF, acute-on-chronic liver failure.

A total of 489 acute decompensated patients with liver cirrhosis were selected in this study (Figure 1). Among these 489 patients (409 patients without ACLF and 80 patients with ACLF), PPI therapy was used in 299 (61.1%) patients: 256/409 (62.6%) patients without ACLF and 43/80 (53.8%) patients with ACLF (Figure 1; Table 1). The PPIs included omeprazole (*n* = 98; daily dose 40–80 mg), pantoprazole (*n* = 132; daily dose 40–80 mg), and lansoprazole (*n* = 69; daily dose 30–60 mg).

**Table 1:** Baseline characteristics of the enrolled patients receiving and not receiving proton pump inhibitor (PPI) therapy.

	**Before PSM**			**After PSM**		
**Variables**	**Non-PPI group (*n =* 190)**	**PPI group (*n =* 299)**	***P* value**	**Non-PPI group (*n =* 182)**	**PPI group (*n =* 182)**	***P* value**
Age (years)	55.47 ± 13.21	53.60 ± 11.52	0.081	55.48 ± 13.21	52.26 ± 11.17	0.020
Sex (male%)	141 (74.2%)	239 (79.9%)	0.139	135 (74.2%)	157 (86.3%)	0.004
Hypertension (%)	38 (20.0%)	47 (15.7%)	0.224	37 (20.3%)	21 (11.5%)	0.022
**Etiology of cirrhosis**
HBV	108 (56.8%)	188 (62.9%)	0.184	104 (57.1%)	126 (69.2%)	0.017
Alcohol	38 (20.0%)	50 (16.7%)	0.358	36 (19.8%)	28 (15.4%)	0.271
HBV plus alcohol	1 (0.5%)	3 (1.0%)	0.568	1 (0.5%)	2 (1.1%)	0.563
Others	43 (22.6%)	58 (19.4%)	0.389	41 (22.5%)	26 (14.3%)	0.043
**Previous de–compensation events^a^** **(%)**						
Ascites^a^	89 (46.8%)	108 (36.1%)	0.019	87 (47.8%)	52 (28.6%)	0.000
Upper gastrointestinal bleeding^a^	29 (15.3%)	73 (24.4%)	0.015	25 (13.7%)	57 (31.3%)	0.000
Hepatic encephalopathy^a^	12 (6.3%)	10 (3.3%)	0.123	11 (6.0%)	6 (3.3%)	0.215
Bacterial infections^a^	18 (9.5%)	19 (6.4%)	0.204	15 (8.2%)	12 (6.6%)	0.549
Severe liver injury^a^	23 (12.1%)	24 (8.0%)	0.136	22 (12.1%)	12 (6.6%)	0.072
**Complications of cirrhosis during hospital (%)**						
Hepatic encephalopathy	24 (12.6%)	26 (8.7%)	0.162	20 (11.0%)	16 (8.8%)	0.483
SBP	9 (4.7%)	12 (4.0%)	0.701	9 (4.9%)	9 (4.9%)	1.000
**MELD score**	14.81 ± 8.34	13.10 ± 8.22	0.017	14.81 ± 8.34	11.42 ± 8.20	0.000
**Laboratory data**
Leukocyte count (10^9^/L)	4.4 (2.7–6.6)	4.7 (2.8–7.4)	0.693	4.4 (2.7–6.6)	4.8 (2.6–8.2)	0.652
Platelet count (10^9^/L)	68 (41–103)	71 (45–108)	0.372	68 (41–103)	73 (47–116)	0.150
Hemoglobin (g/L)	105 (90–122)	94 (74–113)	0.000	105 (90–122)	80 (68–99)	0.000
Serum bilirubin (μmol/L)	48 (22–185)	35 (19–138)	0.041	48 (22–185)	28 (17–117)	0.000
Albumin (g/L)	29.1 (25.8–32.5)	29.3 (25.6–33.0)	0.650	29.1 (25.8–32.5)	30.2 (26.9–33.4)	0.114
AST (IU/L)	59 (33–101)	47 (29–91)	0.075	59 (33–101)	40 (27–73)	0.002
ALT (IU/L)	35 (19–66)	26 (17–51)	0.026	35 (19–66)	24 (15–46)	0.006
INR	1.4 (1.2–1.7)	1.3 (1.2–1.6)	0.007	1.4 (1.2–1.7)	1.3 (1.2–1.6)	0.000
Creatinine (μmol/L)	71 (59–93)	73 (59–92)	0.753	71 (59–93)	73 (58–88)	0.778
Serum sodium (mmol/L)	139 (136–141)	139 (136–142)	0.069	139 (136–141)	140 (136–142)	0.000
**CLIF–SOFA score**	5.08 ± 2.60	4.85 ± 2.71	0.224	5.09 ±2.55	4.60 ± 2.89	0.017
**28–day mortality**	25 (13.2%)	32 (10.7%)	0.902	23 (12.6%)	17 (9.3%)	0.681
**90–day mortality**	36 (18.9%)	59 (19.7%)	0.439	34 (18.7%)	32 (17.6%)	0.836

*Data are expressed as mean ± SD, median (Q1–Q3), or number (percentage). Comparisons between the groups were performed by the Student's t-test, the Mann–Whitney U test, or the chi-squared test*.

a *Within the prior 3 months before the hospital admission related to the study. AST, aspartate aminotransferase; ALT, alanine aminotransferase; HBV, hepatitis B virus; INR, international normalized ratio; MELD score, model for end-stage liver disease score; SBP, spontaneous bacterial peritonitis*.

Proton pump inhibitor non-users (*n* = 190) and users (*n* = 299) did not differ significantly with respect to the baseline features (hepatitis B, 56.8 vs. 62.9%); (alcohol abuse, 20.0 vs. 16.7%); age (55.47 ± 13.21 vs. 53.60 ± 11.52 years); gender composition (male, 74.2 vs. 79.9%); prevalence of hypertension (20.0 vs. 15.7%); and history of HE (6.3 vs. 3.3%), bacterial infections (9.5 vs. 6.4%), or severe liver injury (12.1 vs. 8.0%) in the preceding 3 months. Gastrointestinal bleeding in the 3 months prior to admission was significantly more common in PPI users than in nonusers (24.4 vs. 15.3%, *p* = 0.015), while ascites in the 3 months prior to admission was significantly less common in PPI users (36.1 vs. 46.8%, *p* = 0.019). The incidence of HE (8.7 vs. 12.6%) and SBP (4.0 vs. 4.7%) during hospitalization was not differ significantly between PPI users and nonusers (Table 1). PPI users had significantly lower hemoglobin, serum bilirubin, ALT, INR, and the MELD score (*p* = 0, *p* = 0.041, *p* = 0.026, *p* = 0.007, and *p* = 0.017, respectively) (Table 1).

Then, we matched 489 patients with acute decompensated cirrhosis with 1:1. After PSM, there are 182 patients in PPI user group and non-user group, respectively (Table 1). Compared with the decompensated cirrhosis patients before PSM, some baseline characteristics have changed. PPI users were younger than nonusers (52.26 ± 11.17 vs. 55.48 ± 13.21 years, *p* = 0.020) included more male individuals (86.3 vs. 74.2%, *p* = 0.004) and fewer hypertension patients (11.5 vs. 20.3%, *p* = 0.022). Among PPI users, decompensated cirrhosis was more commonly caused by hepatitis B virus alone compared to PPI non-users (69.2 vs. 57.1%, *p* = 0.005) other than by other causes (14.3 vs. 22.5%, *p* = 0.043). In addition, PPI users had remarkably higher serum sodium level (*p* = 0.000), lower AST level, and the CLIF-SOFA score (*p* = 0.002 and *p* = 0.017). Other baseline characteristics did not change significantly.

We also analyzed 80 patients with ACLF in detail and matched them by the same method as above (Table 2). Among patients with ACLF, there were no significant differences between PPI users and non-users in mean age, gender composition, prevalence of hypertension, etiology of cirrhosis, incidence of previous decompensation, complications of cirrhosis during hospital stay, laboratory data, and scores of the MELD and the CLIF-SOFA. Similarly, PPI users and non-users had comparable 28-day mortality (27.8 vs. 45.9%, 0.482) and 90-day mortality rates (47.2 vs. 56.8%, 0.887). After PSM, other features did not change significantly, except for the lower prevalence of hypertension in PPI users (25.0 vs. 5.6%, *p* = 0.023).

**Table 2:** Baseline characteristics of ACLF patients receiving and not receiving PPI therapy.

	**Before PSM**			**After PSM**		
**Variables**	**Non-PPI group (*n =* 37)**	**PPI group (*n =* 43)**	**P value**	**Non-PPI group (*n =* 36)**	**PPI group (*n =* 36)**	**P value**
Age (years)	51.19 ± 11.87	51.07 ± 12.67	0.896	51.19 ± 11.87	52.58 ± 12.59	0.673
Sex (male%)	31 (83.8%)	36 (83.7%)	0.994	30 (83.3%)	31 (86.1%)	0.745
Hypertension (%)	9 (24.3%)	5 (11.6%)	0.139	9 (25.0%)	2 (5.6%)	0.023
**Etiology of cirrhosis**
HBV	27 (73.0%)	30 (69.8%)	0.754	26 (72.2%)	25 (69.4%)	0.797
Alcohol	3 (8.1%)	6 (14.0%)	0.412	3 (8.3%)	6 (16.7%)	0.288
HBV plus alcohol	0	0	1.000	0	0	1.000
Others	7 (18.9%)	7 (16.3%)	0.758	7 (19.4%)	5 (13.9%)	0.530
**Previous de–compensation events^a^** **(%)**						
Ascites^a^	17 (45.9%)	23 (53.5%)	0.504	16 (44.4%)	20 (55.6%)	0.349
Upper gastrointestinal bleeding^a^	3 (8.1%)	1 (2.3%)	0.240	2 (5.6%)	1 (2.8%)	0.558
Hepatic encephalopathy^a^	3 (8.1%)	1 (2.3%)	0.240	3 (8.3%)	1 (2.8%)	0.307
Bacterial infections^a^	2 (5.4%)	3 (7.0%)	0.774	1 (2.8%)	3 (8.3%)	0.307
Severe liver injury^a^	14 (37.8%)	12 (27.9%)	0.347	14 (38.9%)	10 (27.8%)	0.321
**Complications of cirrhosis during hospital (%)**						
Hepatic encephalopathy	6 (16.2%)	6 (14.0%)	0.779	5 (13.9%)	6 (16.7%)	0.745
SBP	1 (2.7%)	2 (4.7%)	0.649	1 (2.8%)	2 (5.6%)	0.558
**MELD score**	25.32 ± 5.79	25.86 ± 6.58	0.945	25.32 ± 5.79	25.48 ± 6.28	0.964
**Laboratory data**
Leukocyte count (10^9^/L)	6.5 (4.7–9.4)	6.9 (3.8–10.8)	0.753	6.5 (4.7–9.4)	6.5 (3.9–9.7)	0.685
Platelet count (10^9^/L)	82 (52–124)	62 (43–100)	0.208	82 (52–124)	62 (42–103)	0.235
Hemoglobin (g/L)	117 (97–142)	109 (98–128)	0.194	117 (97–141)	108 (91–127)	0.112
Serum bilirubin (μmol/L)	391 (275–485)	396 (294–495)	0.579	391 (279–485)	396 (291–497)	0.744
Albumin (g/L)	31.9 (28.5–33.3)	30.0 (26.4–33.9)	0.320	31.9 (28.5–33.3)	29.4 (25.6–33.3)	0.087
AST (IU/L)	139 (91–275)	118 (64–219)	0.316	139 (91–275)	178 (66–217)	0.292
ALT (IU/L)	105 (66–306)	71 (27–204)	0.075	105 (66–306)	78 (26–184)	0.066
INR	2.0 (1.7–2.4)	2.2 (1.6–2.6)	0.954	2 (1.7–2.4)	2.0 (1.6–2.5)	0.665
Creatinine (μmol/L)	65 (57–95)	70 (57–103)	0.810	65 (57–95)	73 (56–115)	0.510
Serum sodium (mmol/L)	137 (134–140)	136 (132–139)	0.539	137 (134–140)	137 (132–139)	0.923
**CLIF–SOFA score**	8.33 ± 1.39	8.90 ± 1.97	0.400	8.33 ± 1.39	9.08 ± 2.05	0.121
**28–day mortality**	17 (45.9%)	12 (27.9%)	0.482	17 (45.9%)	10 (27.8%)	0.091
**90–day mortality**	21 (56.8%)	19 (44.2%)	0.887	21 (56.8%)	17 (47.2%)	0.348

*Data are expressed as mean ± SD, median (Q1–Q3), or number (percentage). Comparisons between the groups were performed by using the Student's t-test, the Mann–Whitney U test, or the chi-squared test*.

a *Within the prior 3 months before the hospital admission related to the study. ACLF, acute-on-chronic liver failure; ALT, alanine aminotransferase; AST, aspartate aminotransferase; CLIF-SOAF score, chronic liver failure-sequential organ failure assessment score; HBV, hepatitis B virus; INR, international normalized ratio; MELD score, model for end-stage liver disease score*.

### Proton Pump Inhibitor Therapy and the Complications of Cirrhosis During Hospitalization

The proportion of patients developing HE during hospitalization was comparable between PPI users and nonusers before and after PSM (8.7 vs. 12.6%, *p* = 0.162; 8.8 vs. 11.0%, *p* = 0.483) (Table 1). This was true even when patients with ACLF (14.0 vs. 16.2%, *p* = 0.779; 16.7 vs. 13.9%, *p* = 0.745) (Table 2). On the multivariate analysis, age [odds ratio (OR) = 1.07, 95% CI: 1.03–1.12, *p* = 0.001], albumin (OR = 1.13, 95% CI: 1.04–1.24, *p* = 0.006), history of previous HE (OR = 242.52, 95% CI: 40.17–1464.11, *p* < 0.001) and the CLIF-SOFA score (OR =2.89, 95% CI: 2.11–3.96, *p* < 0.001) were significantly associated with the risk of developing HE in decompensated cirrhosis patients during hospitalization (Table 3).

**Table 3:** Risk factors associated with HE and SBP in decompensated cirrhosis patients.

**Variables**	**OR**	**95%CI**	***P* value**
**Risk of HE**
Age	1.07	1.03–1.12	0.001
Albumin	1.13	1.04–1.24	0.006
Previous HE^a^	242.52	40.17–1464.11	<0.001
CLIF–SOFA	2.89	2.11–3.96	<0.001
**Risk of SBP**
Previous severe liver injury^a^	3.43	1.16–10.17	0.026
Previous bacterial infections^a^	6.47	2.29–18.29	<0.001

*Statistical analysis was performed by using the logistic regression analysis. For HE and SBP, the variables entered into the multivariate analysis were gender, age, previous ascites, previous upper gastrointestinal bleeding, previous hepatic encephalopathy (HE), previous bacterial infections, previous severe liver injury, the MELD score, leukocyte count, platelet count, hemoglobin, serum bilirubin, albumin, AST, ALT, INR, creatinine, serum sodium, the CLIF-SOFA score, and PPI use*.

a *Within the prior 3 months before the hospital admission related to the study. OR, odds ratio; SBP, spontaneous bacterial peritonitis*.

The proportion of patients developing SBP during hospitalization was comparable between PPI users and nonusers (4.0 vs. 4.7%, *p* = 0.701; 4.9 vs. 4.9%, *p* = 1) (Table 1). This was true even when patients with ACLF were considered separately (4.0 vs. 2.7%, *p* = 0.649; 5.6 vs. 2.8%, *p* = 0.558) (Table 2). On the multivariate analysis, history of previous severe liver injury (OR = 3.43, 95% CI: 1.16–10.17, *p* = 0.026) and previous bacterial infections (OR = 6.47, 95% CI: 2.29–18.29, *p* < 0.001) were independent risk factors for SBP in patients with decompensated cirrhosis (Table 3).

**Figure 2:**
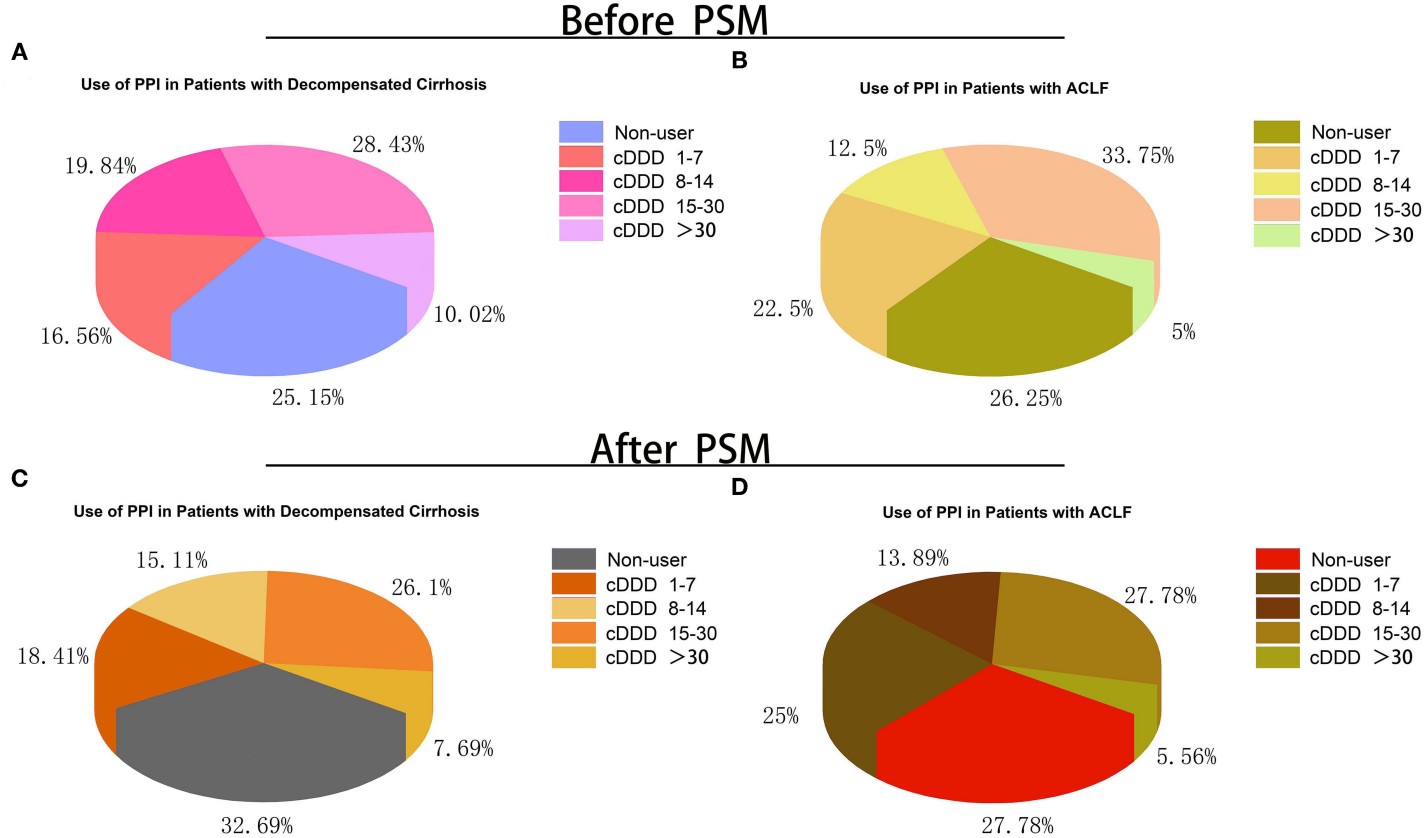
Proportion of patients with different doses and without PPI treatment during hospitalization. **(A)** Proportion of patients with different doses and without PPI treatment in acute decompensated cirrhosis before PSM; **(B)** Proportion of patients with different doses and without PPI treatment in ACLF before PSM; **(C)** Proportion of patients with different doses and without PPI treatment in acute decompensated cirrhosis after PSM; **(D)** Proportion of patients with different doses and without PPI treatment in ACLF after PSM. cDDD, cumulative defined daily dose; PPI, proton pump inhibitor; ACLF, acute-on-chronic liver failure; PSM, propensity score matching.

The cDDD of PPI in patients with decompensated cirrhosis and ACLF is summarized in Figure 2. The cDDDs of PPI in patients with decompensated cirrhosis and ACLF were divided into four groups: 1–7, 8–14, 15–30, and >30 patients. Among all the patients with decompensated cirrhosis, 25.15% were not treated with PPI. Especially, among all the patients with ACLF, 26.25% were not treated with PPI. Before and after PSM, the group of cDDD 15–30 patients accounted for the highest proportion of PPI users in both the decompensated cirrhosis group and ACLF group. But, the proportion of patients with cDDD > 30 patients was the smallest.

**Figure 3:**
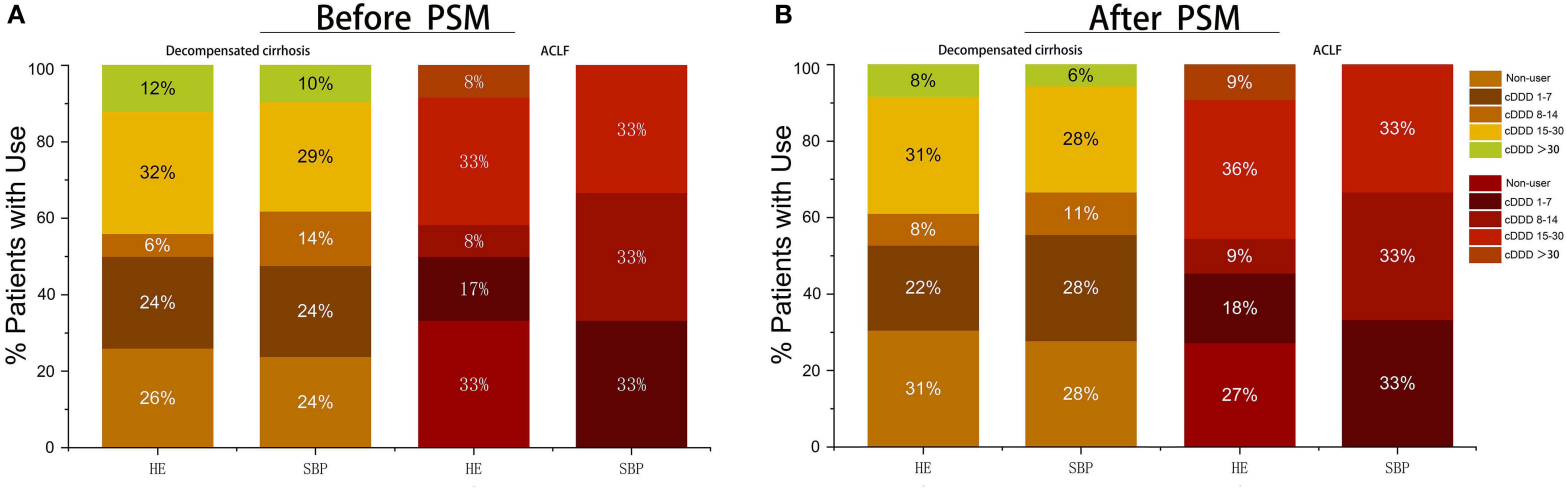
Proportion of patients with different doses and without PPI treatment who suffer from complications during hospitalization. **(A)** Proportion of patients with different doses and without PPI treatment before PSM; **(B)** Proportion of patients with different doses and without PPI treatment after PSM. cDDD, cumulative defined daily dose; PPI, proton pump inhibitor; ACLF, acute-on-chronic liver failure.

Then, we further discussed the relationship between the patients with HE and SBP during hospitalization and the cDDD of PPI (Figure 3). Before PSM, cDDD of 32% patients with HE and cDDD of 29% patients with SBP in decompensated cirrhosis were 15–30 patients, which had the highest proportion. The PPI non-user and cDDD 15–30 patients complicated with HE accounted for a high proportion of 33% in ACLF. After PSM of patients with decompensated cirrhosis, the PPI non-users and cDDD 15–30 patients complicated with HE accounted for a high proportion of 31%, while cDDD 1–7 patients and cDDD 15–30 patients with SBP accounted for a high proportion of 28%. ACLF patients with SBP had the same proportion in the three cDDD groups (1–7, 8–14, and 15–30 patients). Meanwhile, the type and dose of the PPI used were not related to the risk of developing HE or SBP (Table 3).

### Risk Factors for Survival

After median follow-up of 23 months (IQR 6–26 months), the 28-day and 90-day mortality rates were 11.7% (57/489) and 19.4% (95/489), respectively (Table 1; Figure 1).

**Figure 4:**
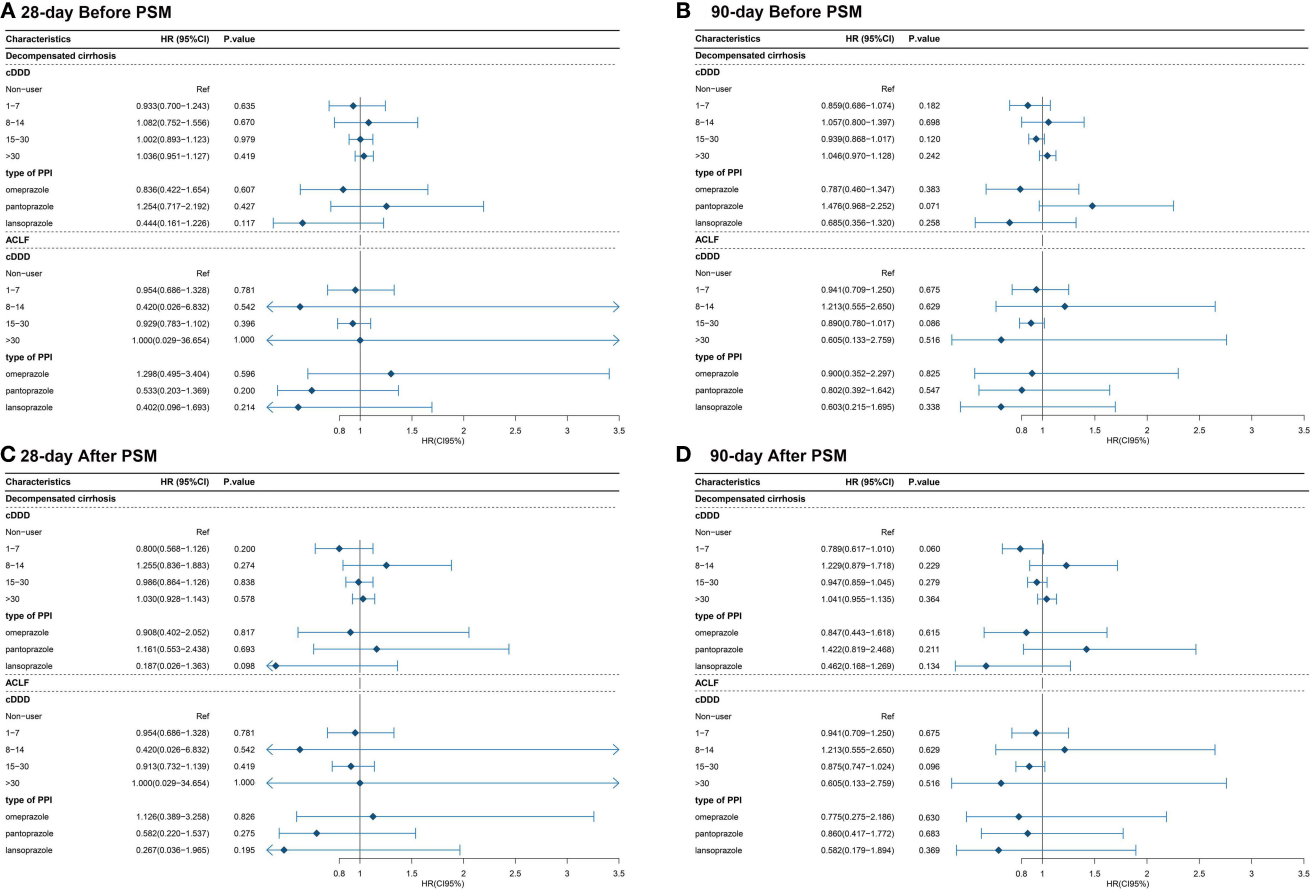
The relationship between the type and dose of the PPI therapy and 28-day and 90-day mortality **(A)** The relationship between the type and dose of the PPI therapy and 28-day mortality before PSM; **(B)** The relationship between the type and dose of the PPI therapy and 90-day mortality before PSM; **(C)** The relationship between the type and dose of the PPI therapy and 28-day mortality after PSM; **(D)** The relationship between the type and dose of the PPI therapy and 90-day mortality after PSM. cDDD, cumulative defined daily dose; PPI, proton pump inhibitor; ACLF, acute-on-chronic liver failure.

**Figure 5:**
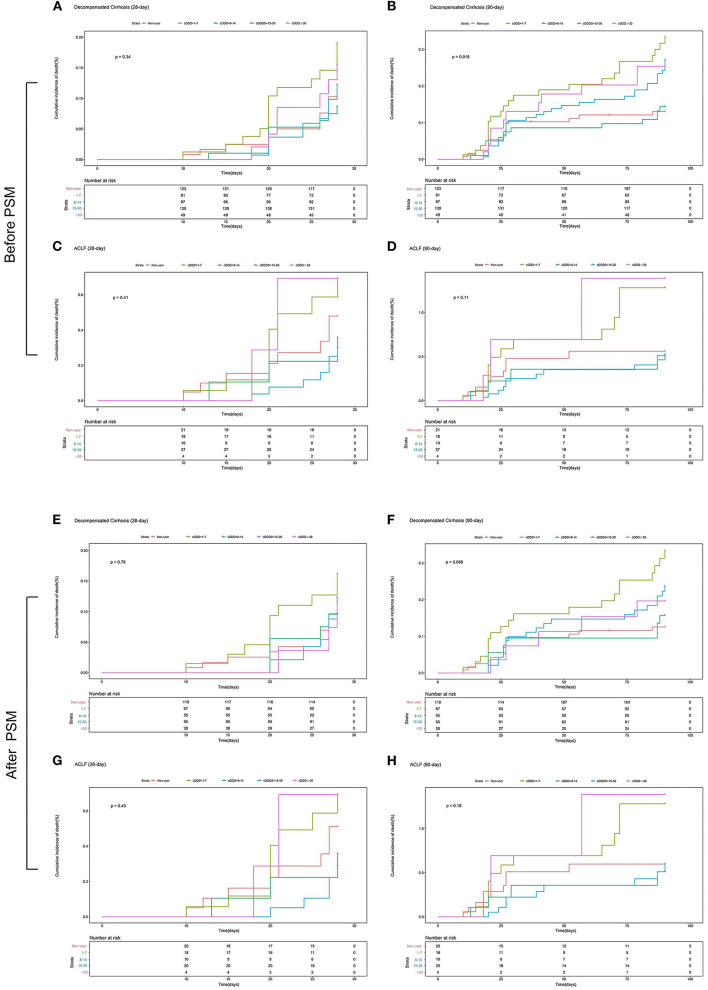
The 28-day or 90-day cumulative incidence of death of patients with and without ACLF with different doses or without PPI treatment during hospitalization. **(A)** The 28-day cumulative incidence of death of patients with decompensated cirrhosis with different doses or without PPI treatment before PSM; **(B)** The 90-day cumulative incidence of death of patients with decompensated cirrhosis with different doses or without PPI treatment before PSM; **(C)** The 28-day cumulative incidence of death of patients with ACLF with different doses or without PPI treatment before PSM; **(D)** The 90-day cumulative incidence of death of patients with ACLF with different doses or without PPI treatment before PSM; **(E)** The 28-day cumulative incidence of death of patients with decompensated cirrhosis with different doses or without PPI treatment after PSM; **(F)** The 90-day cumulative incidence of death of patients with decompensated cirrhosis with different doses or without PPI treatment after PSM; **(G)** The 28-day cumulative incidence of death of patients with ACLF with different doses or without PPI treatment after PSM; **(H)** The 90-day cumulative incidence of death of patients with ACLF with different doses or without PPI treatment after PSM. cDDD, cumulative defined daily dose; PPI, proton pump inhibitor; ACLF, acute-on-chronic liver failure.

The multivariate Cox proportional hazards regression model did not show significant association between the type and dose of the PPI and 28-day and 90-day mortality (Table 4; Figures 4, 5). The *p*-value of the cumulative incidence of mortality death between patients with decompensated liver cirrhosis and the dose of the PPI was 0.018 before PSM, but after PSM, there was no statistical significance. However, age, leukocyte count, and the high MELD score were independent predictors of 28-day and 90-day mortality in cirrhosis patients with AD (Table 4). In addition, previous severe liver injury was an independent predictor of 28-day mortality (Table 4).

**Table 4:** Risk factors associated with mortality in decompensated cirrhosis patients.

**Variables**	**HR**	**95%CI**	***P* value**
**28-day**			
Age	1.04	1.01–1.07	0.007
Leucocyte count	1.07	1.00–1.15	0.042
Previous severe liver injury^a^	2.07	1.17–3.64	0.012
MELD	1.11	1.07–1.16	<0.001
**90-day**			
Age	1.04	1.01–1.06	0.002
Leucocyte count	1.13	1.05–1.21	<0.001
MELD	1.11	1.07–1.15	<0.001

*Statistical analysis was performed by using the Cox proportional hazards regression model. For mortality, variables entered into the multivariate Cox proportional hazards regression model were gender, age, previous ascites, previous upper gastrointestinal bleeding, previous HE, previous bacterial infections, previous severe liver injury, the MELD score, leukocyte count, platelet count, hemoglobin, serum bilirubin, albumin, AST, ALT, INR, creatinine, serum sodium, the CLIF-SOFA score, and PPI use*.

a *Within the prior 3 months before the hospital admission related to the study. ACLF, acute-on-chronic liver failure; HR, hazard ratio; MELD score, model for end-stage liver disease score; INR, international normalized ratio*.

## Discussion

This study aimed to investigate the impact of PPI therapy on complications and prognosis in cirrhosis patients with and without ACLF. PPI therapy is used in as much as 46–78% of cirrhosis patients (7). In this study, PPI was used in 61.1% of cirrhosis patients including 62.6% of patients without ACLF and 53.8% of patients with ACLF. Thus, consistent with previous studies, our results also demonstrate that PPI use is common in cirrhosis patients with and without ACLF (2). The proportions of patients with history of previous gastrointestinal bleeding and low hemoglobin were higher among PPI users than among non-users. This is not surprising because PPIs are commonly used in cirrhosis patients with gastrointestinal bleeding (2, 26).

The type of PPI and the dose were not associated with the risk of HE and SBP. Previous reports have indicated that treatment with acid-suppressing drugs increase the incidence of SBP and HE (7, 11, 14). The explanation offered was that PPIs might promote small intestinal bacterial overgrowth with subsequent bacterial translocation (7, 27). Another explanation was that PPIs may inhibit the functions of neutrophils and natural killer cells (28). However, the association between PPI and SBP has not been consistently demonstrated (15, 21, 29). In one prospective multicenter study of many cirrhosis patients with AD, PPI use did not increase the risk of SBP (17). Our result is in accordance with the conclusion of an earlier meta-analysis that did not support the association between PPI use and SBP or HE (18).

In this study, age, albumin, history of previous HE, and the CLIF-SOFA score were significantly associated with HE during hospitalization. In addition, previous severe liver injury and previous bacterial infections were shown to be independent risk factors for SBP in cirrhosis patients. A previous study has also shown that history of HE is associated with increased incidence of HE during hospitalization (30).

This study found that older age, high leukocyte count, and the high MELD score independently predicted the 28-day and 90-day mortality in cirrhosis patients with AD. Previous studies have also found that age, leukocyte count, and the MELD score have to be independently associated with the risk of death in cirrhosis (31, 32). In this study, PPI therapy was not associated with the 28-day and 90-day mortality in cirrhosis patients with AD or ACLF. Others have also reported that PPI use does not increase the mortality rate in cirrhosis patients with or without ACLF (16, 18).

In conclusion, PPI therapy does not appear to increase the risk of HE or SBP or to shorten survival in cirrhosis patients with or without ACLF. Further prospective multicenter studies with large samples are necessary to confirm our findings and to clarify how PPI therapy is related to complications and disease progression in cirrhosis.

## Data Availability Statement

The original contributions presented in the study are included in the article/Supplementary Material, further inquiries can be directed to the corresponding author/s.

## Ethics Statement

The studies involving human participants were reviewed and approved by the Ethics Committee of the First Affiliated Hospital, School of Medicine of Zhejiang University. The patients/participants provided their written informed consent to participate in this study. Written informed consent was obtained from the individual(s) for the publication of any potentially identifiable images or data included in this article.

## Author Contributions

JS and HZ conceived and designed the study and draw the manuscript. SS, WY, and RZ collected and analyzed the data. JH, XZ, and MY participated in the data interpretation. SS, HZ, and JS revised the manuscript. All authors contributed to the manuscript and approved the submitted version of the manuscript.

## Funding

This study was supported by the National Natural Science Foundation of China (Grant No. 81971982) and the Special Anti-epidemic Project of Zhejiang Provincial Department of Education (Y202043368).

## Conflict of Interest

The authors declare that the research was conducted in the absence of any commercial or financial relationships that could be construed as a potential conflict of interest. The reviewer QY declared a shared affiliation with the authors to the handling editor at time of review.

## Publisher's Note

All claims expressed in this article are solely those of the authors and do not necessarily represent those of their affiliated organizations, or those of the publisher, the editors and the reviewers. Any product that may be evaluated in this article, or claim that may be made by its manufacturer, is not guaranteed or endorsed by the publisher.
